# Temporal equal and active participation in synchronous collaborative learning: Antecedents and effect for learning

**DOI:** 10.1371/journal.pone.0318122

**Published:** 2025-03-24

**Authors:** Sixiong Peng, Shunsaku Komatsuzaki, Ryota Sen

**Affiliations:** 1 Independent Researcher, Tokyo, Japan; 2 The IDEC Institute, Hiroshima University, Higashi-Hiroshima City, Hiroshima, Japan; 3 Department of Civil Engineering, The University of Tokyo, Bunkyo-ku, Tokyo, Japan; University of Almeria: Universidad de Almeria, SPAIN

## Abstract

For teaching 21st century skills, collaborative learning has been increasingly adopted in educational programs nowadays. However, learners often fail to engage in effective collaboration, which severely deteriorates learning gains. To develop support tools for collaborative learning, participation is one of the promising aspects considering its effect on learning gains and its viability for real-time measurement in modern learning environments. This study quantitatively and qualitatively examined the relationship between temporal equal and active participation and learning gains in synchronous collaboration. Our data included 10 teams that collaboratively learn analogical thinking in university coursework. Our result demonstrated the positive relationship between temporal equal and active participation and team learning gains. Detailed observation revealed that equal and active participation often reflected joint information processing which in turn affected learning gains, although this relationship depends on the discussion contents. The effect could be direct, indirect or absent depending on discussion topics and other factors. Our comparative analysis also proposed three antecedents for equal and active participation; maintaining a shared understanding of what to discuss and why to discuss, critical comments for extending discussion, and arguing without completion. We lastly summarized our theoretical implications for equal and active participation and practical implications for supporting collaborative learning.

## 1. Introduction

For teaching 21st century skills, designing opportunities of effective collaborative learning is crucial. Collaborative learning can not only allow learners to acquire deeper domain knowledge, but can cultivate their creative and critical thinking skills, increase their learning motivation, and develop their social and communication skills for effective teamwork [1,2]. Designing effective collaborative learning does not just include providing opportunities to collaborate, but also supporting collaboration because learners often fail to engage in effective social interactions [3]. For example, Barron [4] contrasted two groups solving math problems and found that a group with lower written scores lacked shared task alignment, joint attention, and mutuality. When interaction is mediated by computer, social interaction is even harder to achieve. Several studies showed only one or two notes per week were posted in discussion forums [5,6]. To support collaborative learning, teachers need to prepare a learning environment as well as provide real-time intervention [7].

Participation is one of the key aspects of collaborative learning that is useful for analyzing and supporting collaborative learning [8]. Participation serves as a vehicle for learners to share and revise their skill and knowledge and regulate team learning [9–11]. Students with higher participation tend to achieve higher learning gains [12,13]. At a team level, equal and active participation is considered as desirable [8,14]. Empirical studies suggested members in equal participating teams showed higher learning gains [15] and were more satisfied with the collaboration [10]. Equal and active participation enables learners to potentially build common grounds, socially regulate their learning, or engage in co-construction of knowledge [9,16,17]. Conversely, unequal participation has been constantly reported as a source of dissatisfaction [18,19]. Learners generally have expectations of participation towards other learners, and if this expectation is not met, they would perceive social loafing and feel unfair [10,20]. Therefore, monitoring and ensuring equal and active participation can be an effective strategy to increase the quality of collaborative learning.

Participation is easier to measure objectively than other indicators of team interaction. With the recent trends of introduction of information and communication technology tools in formal and informal learning, it has become more feasible to measure and utilize the participation levels in real time. When learners use social networking services such as Slack, online chats or actions can be collected for calculating the level of participation. Even in synchronous communication, video conferencing tools such as Zoom allow recording voice data without much noise. As for face to face collaboration, recording voice data would be more difficult, but several tools for recording voice data and visualizing participation have been introduced [10,21–23]. This opens opportunities for both students and teachers to monitor and improve the quality of collaborative learning.

Despite its potential, several limitations in the current understanding of equal or active participation have inhibited its effective utilization. First, most of the previous research analyze asynchronous online collaboration (e.g., [10,15,24–26]). This is primarily because communication data in social networking services are more easily available for analysis. However, considering a number of benefits of synchronous communication such as enhancing mutual trust and increasing motivation, analyzing participation in synchronous communication is crucial [27,28]. Because synchronous and asynchronous collaboration differ in amount, contents, and pattern of discussion [29], insights in asynchronous collaboration are not necessarily applicable for synchronous communication. In addition, existing studies often measured the level of participation throughout the whole time of collaboration [30,31]. This inherently viewed participation as a relatively stable group feature. In reality, the level of participation dynamically changes depending on teams’ motivational, emotional, and cognitive states [32–34].

Particularly, temporal equal and active participation in a brief period can be perceived as qualitatively distinct times where all team members are deeply engaged in discussions. Such moments have been described in prior research as regions in which members are highly involved in the discussion [35], moments when interaction flows [36], or group flow [37]. We conceptualize temporal equal and active participation based on the theory of group flow. Group flow is “defined as a shared state of balance within a group as represented by fluent, positive interactions within the group, a high collective competence of the group, and collective state of mind of the group by means of positive relationships between group members.” [38], extending the concept of individual flow states [39] to the group level [40]. Sawyer [40] argues that group flow is a localized phenomenon occurring when all members play equal roles and maintain balanced participation, continuously communicating with one another, which shares similar aspects with temporal equal and active participation. Group flow is also suggested to be a neurologically distinct state, marked by synchronized activity in the left middle temporal cortex (L-MTC) among members [41]. Group flow represents the most creative state of a group, where members intensively share, negotiate, and elaborate on each other’s knowledge. Therefore, it can play a crucial role in constructing and acquiring new knowledge in collaborative learning.

In the context of collaborative learning, we could find a few studies examining temporal participation in synchronous collaboration [i.e., 14,32,42]. Sinha et al [14] conceptualized engagement as temporal, group level, and multi-faceted and illustrated how each aspect of engagement interrelated as group work unfolded. Their detailed examinations of two groups suggested that high behavioral and social engagement, which overlapped with our notion of active and equal participation, led to cognitive and conceptual-to-consequential engagement. Isohätälä et al [32] also adopted a temporal view of participation and found that the level of participation increased when participants engaged in socio-emotional interactions although this tendency was largely influenced by the nature of their task. While these results provided an initial hypothesis of the nature and effects of equal and active participation, further understandings of the antecedents, effect, and moderators of equal and active participation are needed to utilize this indicator for supporting synchronous collaborative learning.

This research investigates temporal equal and active participation in 10 teams in the idea generation workshop for teaching analogical thinking. Guiding research questions are:

- Does temporal, not overall, equal and active participation affect learning gains in a synchronous collaborative learning?
- What are the antecedents of temporal equal and active participation?

## 2. Materials and methods

### 2.1. Participants and workshop process

We analyzed a 2-week online workshop in an innovation study course at the University of Tokyo. The workshop was originally planned for multiple studies, including Sen [43], and students had been informed of possible experiments before they registered for the course. The participants included 47 students of their second to fourth year of bachelor’s course. Nine were female and 38 were male. Participants were divided into 10 teams of 4 to 5 members so that all teams were as homogeneous as possible in terms of their gender, their previous workshop experience, and their major. Having received an ethics approval on this particular research from the university’s research ethics committee, all participants of the workshop above received informed consent using approved documents. None wished to opt out of the study until the designated deadline explained in the informed consent process. The recorded data were thus accessed for this study on 4–8 April 2022. We authors had access to the information that could identify individual participants as it was necessary for our analysis. It had been securely stored as approved by the ethics committee.

**Fig 1:**
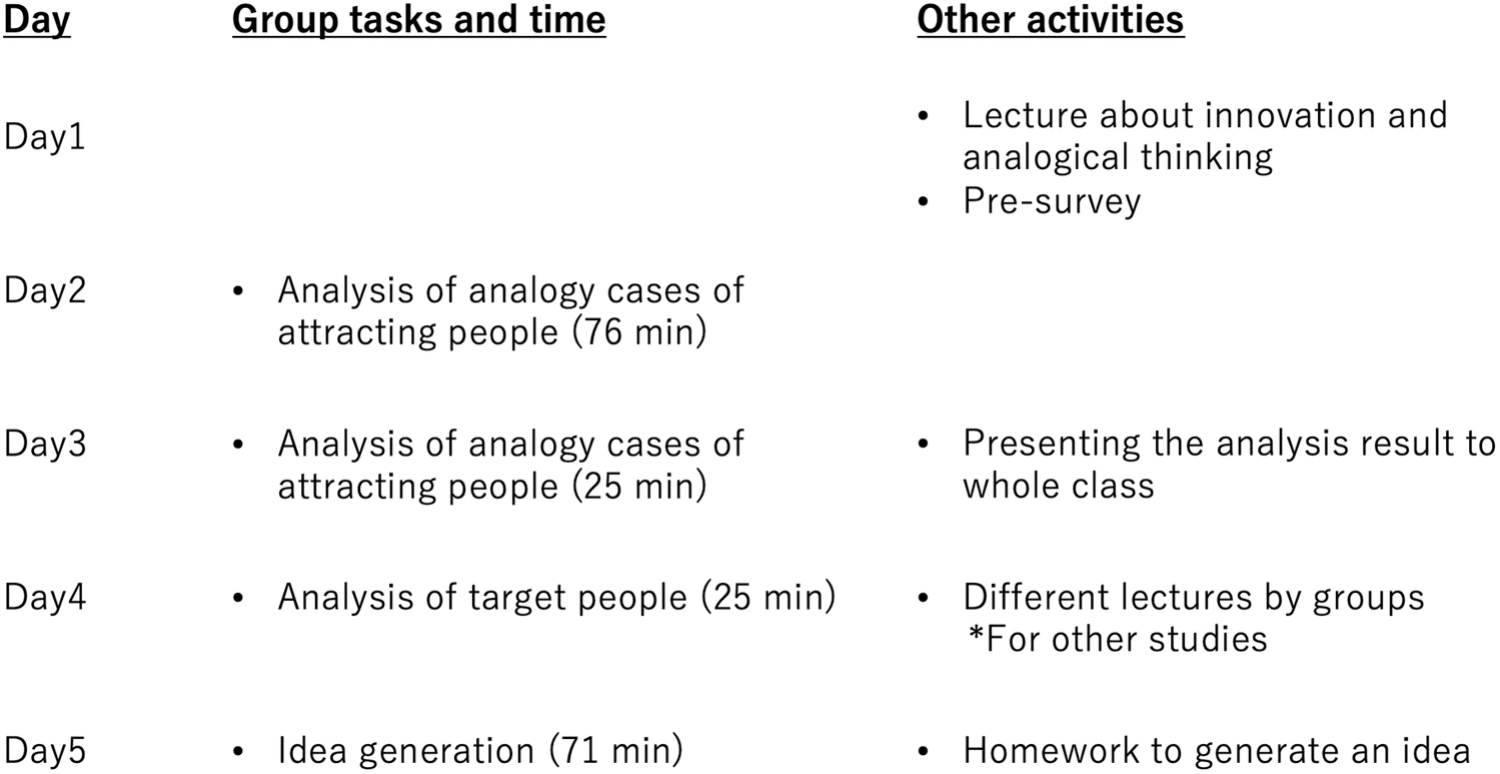
Group tasks and other activities in the workshop.

Participants followed the instruction to generate ideas for increasing diversity in the University of Tokyo using analogical thinking. Fig 1 summarized the group tasks and other activities during the workshop. Whole workshop was conducted online using a video conferencing application “Zoom” and a digital whiteboard collaboration space “Mural”.

Main process of workshops included analyzing analogy cases of attracting people (day 2 and day 3), analyzing target people (day 4), and generating ideas (day 5). Lecturers prepared 18 analogy cases of attracting people such as Pokémon GO, Huis Ten Bosch, and Hometown Tax Donation Program. Lectures on the fourth day were designed for other studies [43]. Four types of lectures were provided to different teams for manipulating the level of understanding of analogical thinking and motivation to utilize it, but this manipulation was a failure. Because experimental groups and control groups show no difference in our variables in this study, we ignore the effect of this lecture.

### 2.2. Dataset

**Fig 2:**
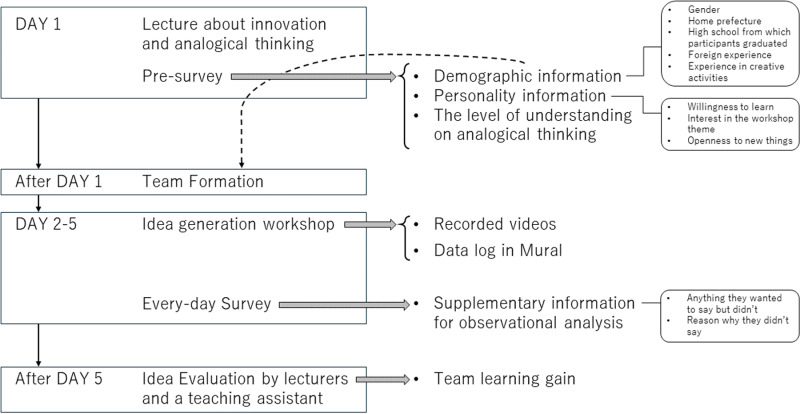
Overall procedure of data collection.

We collected 5 types of data for this study as illustrated in Fig 2: video recordings, data log in Mural, pre-survey, every-day survey, and generated ideas with its evaluation.

#### 2.2.1. Video recording.

We recorded group works via the recording function of Zoom. Recordings of team 4 on day 2, team 6 on day 3, and team 8 on day 4 were missing due to a technical problem. For calculating the level of participation, start time and end time of every utterance that is longer than 0.5 seconds was calculated using a locally developed application. We also used this video recording for observational analysis.

#### 2.2.2. Data log in Mural.

The work result of every team was automatically saved in Mural. We used this information for observational analysis.

#### 2.2.3. Pre-survey.

Pre-survey included demographic questions, personality questions, and 3 questions for measuring understanding of analogical thinking. Each question about analogical thinking provided a pair of cases and asked to choose whether the pairs were structurally similar or not and superficially similar or not. We consider the number of right answers as the level of initial understanding of analogical thinking. All but one participant answered this survey.

#### 2.2.4. Every-day survey.

Every-day survey asked participants whether there was anything they wanted to say but didn’t. They answered the contents they wanted to say and the reason they did not say. Because it can be hard to recall this incident, they were asked to leave a note during the workshop if possible. This question was for obtaining information about the reason for low participation. We used the survey result to support our observational analysis.

#### 2.2.5. Generated ideas with its evaluation.

Participants were instructed to generate one idea and explain it with related analogy cases in their homework of day 5. All ideas were evaluated by two lecturers (evaluator A and B) and one teaching assistant (evaluator C) as to whether they were structurally similar, superficially similar, or unrelated to the analogy case. This evaluation was based on existing research considering structural similarity as an essential part in analogy [44,45] and demonstrating utilization of structural features rather than superficial features resulted in more novel ideas [46]. The interrater agreement was moderate (Fleiss’ Kappa =  0.52). After inspection of the evaluation and discussion with evaluators, evaluation of B was found to be more severe. If his evaluation was excluded, the interrater agreement was substantial (Fleiss’ Kappa =  0.71). Thus, we decided the final evaluation based on the evaluation of A and C. If their evaluation conflicted, we considered evaluation of B and took the most common evaluation.

### 2.3. Analysis

#### 2.3.1. Operationalization of temporal equal and active participation.

To measure temporal level of participation, one should determine the time span for calculating equality and activeness of participation. Most studies adopted a fixed time span around 5 minutes [14,32] while Peng, Amakasu and Horii [47] proposed fluctuating time spans based on changes of communication patterns. We adopt this more adaptive method to operationalize temporal equal and active participation.

**Fig 3:**
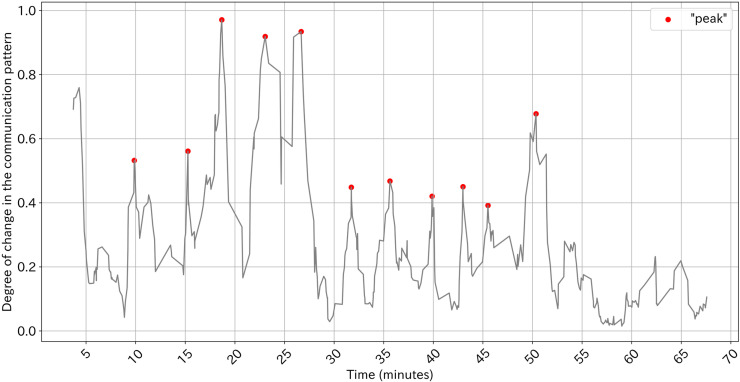
Example of degree of change along with time.

**Detecting the point of large change in communication pattern:** We firstly determine time spans for calculating equality and activeness of participation based on Peng, Amakasu and Horii [47]. For every start time of utterance, we calculated the degree of change in communication pattern (DC) defined as follows:


$$DC\left(t\right)=1-\left(x_{11}^{t-s}...x_{ij}^{t-s}...x_{nn}^{t-s}\right)\cdot \left(x_{11}^{t}...x_{ij}^{t}...x_{nn}^{t}\right)\left(t\geq s\right)$$

where *t* is time; *n* is the number of team members; *x*_*ij*_^*t*^ is the number of turn-taking between speaker *i* and speaker *j* within *t* to *t + s* minutes; s is the time span for comparing communication patterns. *DC(t)* denotes the difference in the pattern of turn-taking before *t* and after *t*. Then we calculated “peak” that satisfies following conditions; the DC at the “peak” is maximal, the DC at the “peak” is over *v*, and there is at least one minimal DC which is smaller than the DC at the “peak” by at least *d* between neighboring “peaks”. The “peak” indicates the point where the communication pattern of the previous s minutes and the subsequent s minutes differ greatly. We used s =  180 (seconds), v =  0.3 and d =  0.2 as parameters. The DC and the peak can be visualized as Fig 3. It demonstrates that there are points of greater change in communication patterns. We consider the peaks and the start time and end time of silence longer than 3 minutes as the borders in which the pattern of turn-taking is relatively stable.

**Calculating equality and activeness of participation:** Between every border, we calculated the activeness of participation as the number of turn-taking, and equality as the formula below.


Activeness=Numberofturntaking

$$Equality=1-CV\_turn/CV\_turn_{max}$$

where *CV_Turn* is coefficient of variation of the number of turn-taking between every pair.

For example, if a team has 3 members and the number of turn-taking between member 1 and member 2 is 4, that between member 2 and member 3 is 6, and that between member 1 and member 3 is 2, Activeness is 12 (4 + 6 + 2). As for equality, CV-Turn is 0.41, which is calculated by 1.63 (standard deviation of 4, 6, and 2) divided by 4 (mean of 4, 6, and 2) and CV-Turn_max_ is 1.41, which is calculated by 0.47 (mean of 1, 0, and 0) divided by 0.33 (mean of 1, 0, and 0). Hence Equality is 0.71 (1 - 0.41/ 1.41). We adopted this value because it could fairly indicate equality of participation when the number of turn-taking varies and the number of members varies, which were drawbacks of existing indicators of participation [48,49].

**Defining an equal and active participating (EAP) section:** Considering neither of the distributions of equality and activeness were standard distribution, we defined an equal and active participating (EAP) section as a section where both equality and activeness are more than their weighted median. We used weighted means because the duration of each section was not the same.

#### 2.3.2. Team learning gain.

As the purpose of the workshop is to learn analogical thinking, we defined team learning gain as the ratio of team members who generated ideas based on structural similarity.

#### 2.3.3. Regression analysis.

We conducted the regression analysis with the ratio of EAP sections during whole teamwork time as an independent variable and the team learning gains as a dependent variable. In our dataset, outsiders sometimes visited a team to give advice. Because the communication pattern with outsiders speaking does not represent the team characteristics, we excluded sections with outsiders speaking time more than 5% of its duration. Because the level of understanding of the members before workshops could affect this, we checked the correlation between the team learning gains and the average number of correct answers in questions about analogical thinking in the pre-survey, but no correlation was found (r = 0.01, p = 0.97). Hence, we did not control this value.

We also conducted additional regression analysis to support our main result. Because EAP sections changed depending on the threshold of equality and activeness, we regressed with stricter threshold (weighted 60th percentile) and looser threshold (weighted 40th percentile). We also regressed with equal participating sections (equality is more than their weighted median) and with active participating sections (activeness is more than their weighted median) to check whether both features were important. Lastly, to compare with previous studies that operationalized equal and active participation in all time, we calculated equality in all time and activeness in all time (divided by total time) and conducted multiple regression with the team learning gain.

#### 2.3.4. Observational analysis.

**Fig 4:**
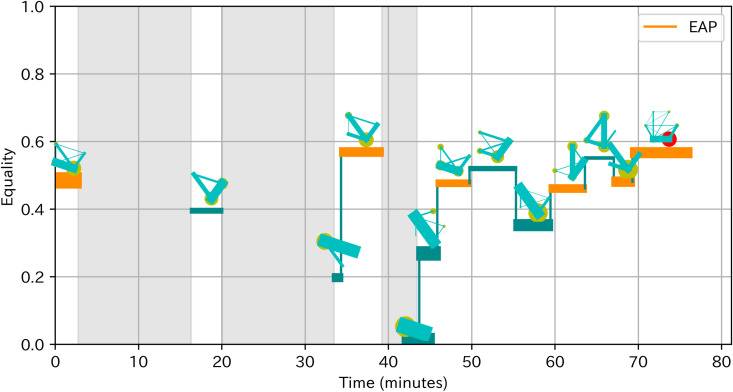
Example of visualization of EAP sections with communication patterns. The horizontal axis is time. The vertical axis is equality. The line width is proportional to activeness. Lines are colored orange in EAP sections. The diagram above the line shows the communication pattern, where the yellow circle indicates speaking time of a member and green line indicates number of turn-taking between two members. An outsider is represented as a red circle. Silence over 3 minutes is colored gray.

To obtain a deeper understanding of the effect and antecedents of EAP sections, we conducted observational analysis for two teams with the highest team learning gain and two teams (team 4 and 8) with the lowest team learning gain (team 5 and 7). We based our analysis on a socio-cognitive framework in which team processes such as EAP sections influence team outcomes through individual or team cognitive processes [50]. Prior to the observation, we revisited the ideas of these teams to familiarize the details of the ideas and related analogy cases. We firstly transcribed the discussion and listed the discussion topics on the day 5 to identify the cognitive processes that could influence the acquisition of analogical thinking. We focused on the day 5 because preliminary observational analysis suggested that the discussion of idea generation on Day 5 most directly influenced the thinking process in later idea generation in the homework. Indeed, members in teams with higher learning gain often created ideas related to analogy cases or idea concepts discussed in teams. Next, we visualized the time of EAP sections with changes of communication patterns as Fig 4 based on the visualizing method of Peng, Amakasu and Horii [47]. These visual representations supported our analysis by not only clarifying the time of EAP sections and depicting the changes of participation leading up to EAP sections, but also providing rich information on participation in teams with fewer EAP sections. Finally, based on this information about discussion topics and changes of participation, we examined how EAP sections related to cognitive processes and learning gains by contrasting teams with higher and lower learning gains. In addition, we examined the process leading to EAP sections as well as comparing teams to obtain insights on antecedents of EAP.

## 3. Results

### 3.1. Results of regression analysis

**Fig 5:**
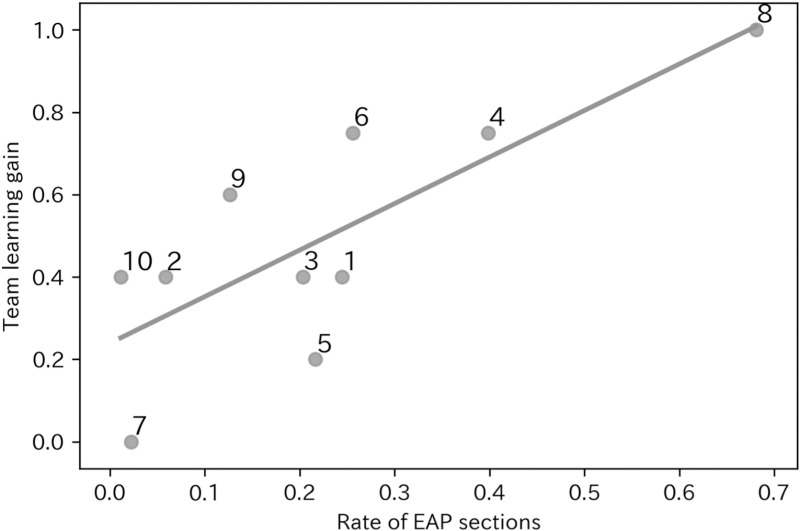
Scatter plot of the rate of EAP sections and the team learning gain.

Fig 5 showed the scatter plot of the rate of EAP sections and the team learning gain. We drew the linear regression and plotted the team number. Regression analysis showed the significant positive regression coefficient (β=1.13, R^2^ = 0.61, p = 0.008). This positive relationship was also found with stricter threshold for EAP (β=1.33, R^2 = 0.62, p = 0.007) and looser threshold (β=0.79, R^2^ = 0.48, p = 0.027), which suggested the stability of the positive relationship. The regression with rate of only equal participating sections were significant (β=0.82, R^2^ = 0.47, p = 0.03), but that with only active participating sections were not significant (β=0.48, R^2^ = 0.12, p = 0.33), which suggested equality played more important role. Lastly, multiple regression with equality in all time and activeness in all time revealed no significant relationships (t = -0.49, p = 0.64 for equality, t = -1.45, p = 0.19 for activeness), which indicated the importance of a temporality of equality and activeness of participation.

### 3.2. Results of observational analysis

We compared 2 teams with the high rate of ideas with structural similarity (team 4 and team 8) and 2 teams with the low rate of ideas with structural similarity (team 5 and team 7).

#### 3.2.1. Team 4.

**Discussion process:** Table 1 showed the discussion topics of team 4. In the first 30 minutes, the team discussed target people to attract, and decided to target students who are willing to enter the University of Tokyo but cannot for some reasons (4.2 - 4.4). Then the team started to discuss which analogy cases can be utilized, but they soon found their understanding of analogy cases was insufficient (4.6). Hence, they started to read case materials and analyzed several cases including “RIZAP”, “Trip Advisor “, and “B1 Grand Prix” (4.7,4.8, 4.11). They then discussed again which analogy cases can be utilized and agreed to focus on “B1 Grand Prix” (4.13). At last, they discussed several ideas based on this case (4.14). This discussion seemed to greatly affect members’ strategy for generating ideas in their homework because all members chose “B1 Grand Prix” as an analogy case while ideas were different. All except one idea were structurally similar to the analogy case.

**Table 1:** Discussion topics of team 4.

Index	Start time	Topic
4.1	0:00:00	What to discuss
4.2	0:03:49	Barriers for students who are willing to enter the University of Tokyo
4.3	0:10:55	Reasons for unwillingness to enter the University of Tokyo
4.4	0:30:13	Which to focus: students who are willing to enter the University of Tokyo or those who are not
4.5	0:31:58	What attracts students who are willing to enter the University of Tokyo
4.6	0:34:50	Which analogy cases can be utilized
4.7	0:44:19	Whether the case “RIZAP” can be utilized
4.8	0:49:19	Whether the case “Trip Advisor” can be utilized
4.9	0:53:37	How to get the case material
4.10	0:54:58	What to discuss
4.11	0:57:09	Whether the case “B1 Grand Prix” can be utilized
4.12	1:01:37	The goal of today
4.13	1:03:20	Which analogy cases can be utilized
4.14	1:04:22	Ideas based on the case “B1 Grand Prix”

**Pattern of turn-taking:**

**Fig 6:**
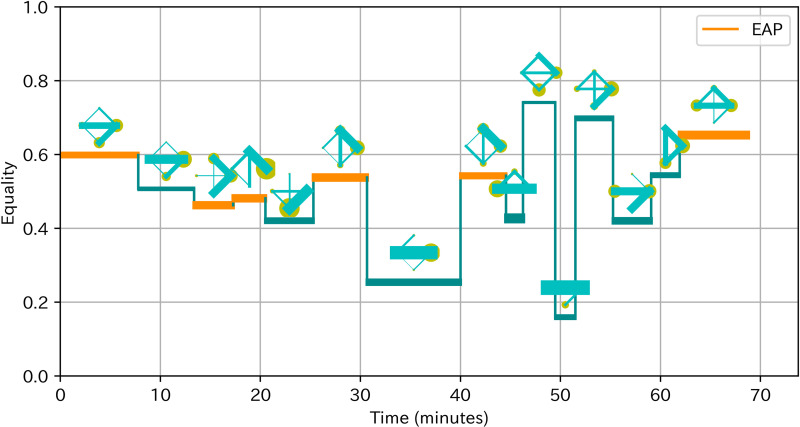
Pattern of turn-taking of team 4.

Fig 6 showed the pattern of turn-taking of team 4. There were five EAP sections. The Following part described our detailed observation, in the order of more obvious relevance to the learning of analogical thinking.

**Fig 7:**
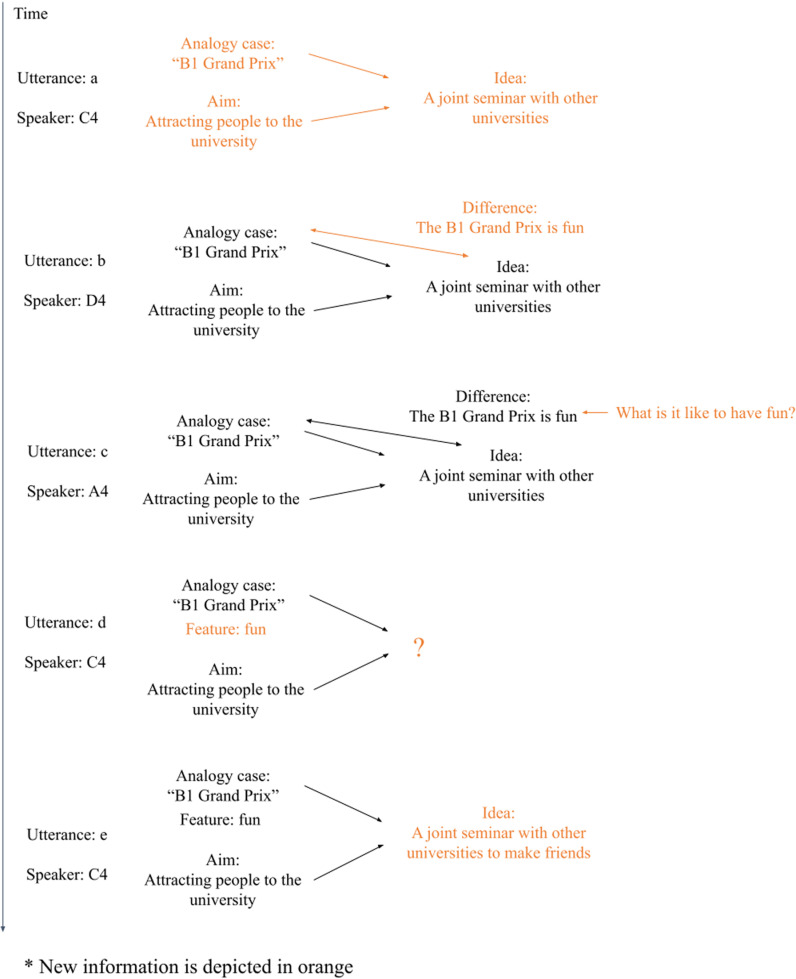
Joint information processing in the fifth EAP section in team 4.

Among these sections, especially the discussion in the fifth EAP section (62 ~  69 minutes) demonstrated the most direct influence on an acquisition of analogical thinking. Table 2 showed the key utterances during the fifth EAP section. After deciding to use the B1 Grand Prix case as a team, C4 firstly suggested an idea, but he noticed that it is only superficially similar to the analogy case (a). Listening to C4’s idea, D4 pointed out characteristics of B1 Grand Prix, which implicitly suggested the drawback of the U’s idea (b). A4 deepened this point by providing another case based on her experience (c). Then C4 verbalized the challenge they are facing (d), which finally led to a solution by D4 (e). This solution was accepted by C4 and the team looked very excited (f). A4 lastly added new features to the idea (g). This joint information processing can be summarized as Fig 7. The initial idea of C4 only utilized superficial similarity of the analogy case, it made D4 realize an important feature of the case by comparing the case and the idea. This feature was elaborated by A4. Then C4 clarified the requirement of the idea finally leading to idea generation based on structural similarity by C4. This process could be considered as a very effective thinking process for generating ideas by analogical thinking, and experiencing it could impact the understanding of analogical thinking, leading to ideas with structural similarity in their homework. Although members could not complete this process alone, they achieved it by complementing each other’s thinking. Equal and active participation captured this joint information processing for analogical thinking.

**Table 2:** Key utterances in the fifth EAP section of team 4.

Index	Start time	Speaker	Utterance
a	1:04:22	C4	Do you know information sessions for job hunting where a number of companies gather at a rented location? If we did a university version of that, it would be like the B1 Grand Prix, but with a superficial similarity. I think it would be good if we can think more deeper in terms of analogy
b	1:05:11	D4	I think the attractiveness of the B1 Grand Prix itself is an important factor. If it was a hassle, I wouldn’t have thought of going.
c	1:05:47	A4	Is It like going to a shopping mall? Or like going to a department store.
d	1:07:00	C4	It’s hard to have fun there and at the same time promote the attractiveness of the university.
e	1:07:09	D4	But it would be nice if I can make friends there. We discussed the importance of the environment, right? We can relate to that.
f	1:07:38	C4	I see. Genius. I see. That sounds great.
g	1:08:07	A4	Also, it would be nice to have more opportunities to meet college students.

The conversation was originally Japanese and translated by the first author.

The first (0 ~  8 minutes) and fourth (40 ~  45 minutes) EAP sections are also related to analogical thinking, but the relationship was more indirect. As for the first EAP section, the topic was mainly about confirmation of their task and what to discuss. Table 3 showed the key utterances. B4 firstly confirmed their previous consensus to target high schoolers in local areas (h) and suggested to find analogy cases related to the target (j). However, A4 shared her concern of insufficient understanding of problems of the target (k). Because this statement was made without clearly relating to B4’s suggestion, B4 had to relate A4’s concern to his thoughts. Then B4 interpreted A4’s statement as a statement related to the way of searching analogy cases and shared a revised strategy of searching analogy cases with focusing on the problem structure (m). This was accepted by the team, and C4 further suggested dividing the target into two based on the difference in problem structure (n). This series of discussions resulted in the creation of a task procedure of analyzing the structure of the problem for the two target groups and searching analogy cases that relate to the structure of the problem. This procedure, which was created by a joint information processing similar to discussion in the fifth EAP, was more elaborated than the initial procedure suggested by B4, and could be convincing to the members because it reflected everyone’s statement. Although its contribution to the learning of analogical thinking was more unclear, it could indirectly influence the learning of analogical thinking considering this procedure made teams focus on structure of the problem and analogy cases.

**Table 3:** Key utterances in the first EAP section of team 4.

Index	Start time	Speaker	Utterance
h	0:00:56	B4	Are our target people local high school students?
i	0:01:06	A4	Yes, that’s what we were talking about.
j	0:01:12	B4	Let’s see any of 18 cases are related to that
k	0:01:19	A4	Maybe we should analyze the cause and consequence of the problem, not just focusing on locality as a problem?
l	0:02:13	C4	Yes, that’s also possible.
m	0:02:57	B4	I see. You mean we should search for cases that have the same problem structures.
n	0:03:17	C4	Agree. When I tried to think of a structure of the problem, I wrote it down in the lower left as a memo. I felt like the problem could be divided into at least two parts, those who give up trying to enter Tokyo University and those who don’t try to enter in the first place.

The conversation was originally Japanese and translated by the first author.

As for the fourth section, the topic was about how to search appropriate analogy cases. Table 4 showed the key utterances. The team had difficulty in searching analogy cases at first as expressed in B4’s utterance (i). For making search easier, A4 suggested an alternative searching strategy (p). Although this idea of changing searching strategy seemed to be effective, A4’s specific strategy seemed to be inappropriate. Thus, D4 pointed out this fallacy (q) and corrected the searching strategy (r). Listening to D4’s statement, B4 introduced a useful analogy case with the reason. Given that B4 firstly could not effectively search an analogy case, the discussion certainly affected B4’s understanding of the way to utilize the analogy cases. Again, this process is similar to the discussion of the fifth EAP in terms of jointly experiencing effective information processing for using analogy cases.

**Table 4:** Key utterances in the fourth EAP section of team 4.

Index	Start time	Speaker	Utterance
o	0:40:19	B4	I don’t know if any of these fit... it’s hard.
p	0:40:28	A4	It’s hard, isn’t it? It may be difficult to think of something that would fit, but how about thinking of something that is attractive but not reaching the target audience, or the reason for not reaching the target audience. For example, it could be something invisible because it is covered by some biases, or something that is not being communicated enough.
q	0:41:34	D4	That is like the second target, isn’t it?
r	0:41:53	D4	We should rather think about those who want to enter but don’t know how to approach it, right?
s	0:42:55	B4	I feel the Hometown Tax Donation Program is a way to give back or support the hometown to people who did not know how to do so.

The conversation was originally Japanese and translated by the first author.

For the second (13 ~  21 minutes) and third (25 ~  31 minutes) EAP segments, their relationship with analogical thinking was unclear. In the second EAP, everyone confirmed that the serious impression of the University of Tokyo demotivated students to enter it by sharing their experiences and opinions, followed by skepticism about the significance of breaking the serious impression. Similarly, in the third EAP, everyone confirmed that the university was often compared with medical schools, but skepticism was expressed about the legitimacy to change students’ willingness. Overall, in these discussions, EAP basically captured the situations that everyone agrees on a certain topic. The relationship between the content of these discussions and the analogical thinking was unclear.

#### 3.2.2. Team 8.

**Discussion process:** Table 5 showed the discussion topics of team 8. The team spent only a short time discussing target people (8.3) and moved to analyzing analogy cases. They analyzed more than 7 cases (8.6, 8.8–8.10, 8.12, 8.14) with sometimes focusing on ideas generated during the case analysis (8.7, 8.13). They lastly summarized their analysis result (8.15). As well as other parts, the discussion in 8.13 greatly could affect idea generation in their homework because 3 out of 4 members generated similar ideas as the idea discussed in 8.13. All their ideas were structurally similar to the analogy case.

**Table 5:** Discussion topics of team 8.

Index	Start time	Topic
8.1	0:01:01	What to discuss
8.2	0:04:32	Possibility to use analogy cases for generating ideas
8.3	0:07:09	Who should be targeted
8.4	0:10:42	What to discuss
8.5	0:14:24	Whether the case about security can be utilized
8.6	0:17:55	Whether the case “Tanita Cafe” can be utilized
8.7	0:31:30	Idea of books written by students at the University of Tokyo
8.8	0:40:33	Whether the case “Rule Change of Judo” can be utilized
8.9	0:43:47	Whether the case “Hometown Tax Donation Program” can be utilized
8.10	0:48:48	Whether the case “Kesennuma Knitting” can be utilized
8.11	0:53:44	The goal of today
8.12	0:54:43	Whether the case “Pokémon GO” can be utilized
8.13	0:58:25	Idea of joint seminars by multiple university
8.14	1:04:17	Whether the case “Randoseru School Bag” can be utilized
8.15	1:08:45	Summary of the analysis result

**Pattern of turn-taking:**

**Fig 8:**
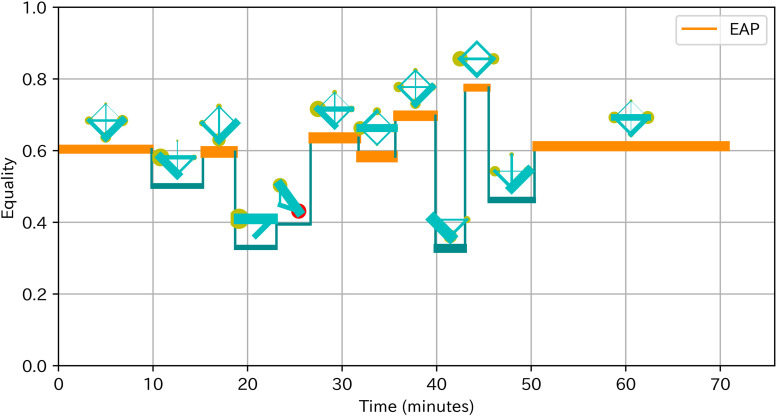
Pattern of turn-taking of team 8.

Fig 8 showed the pattern of turn-taking of team 8. There were five equal participating sections. The Following part described our detailed observation, in the order of more obvious relevance to the learning of analogical thinking.

The fifth EAP section (50 ~  71 minutes) included the most influencing topics 8.13. Table 6 showed the key utterances in the fifth section. In the analysis of the case Pokémon GO, D8 argued that the desire to collect things is the key mechanism of Pokémon GO (t). After D8 elaborated it, C8 asked the ideas if it was applied to the University of Tokyo (u). D8 answered with wild ideas (v), but then C8 suggested a new more realistic idea (w). This idea was well accepted by other members. B8 indeed pointed out other strengths of the idea (x). C8 later pointed out the drawback of the idea (y) and he suggested major modification to the idea to overcome the drawback based on another analogy case (z). This process of information processing is an effective use of analogical thinking. Following D8’s interpretation of the mechanisms that attract people in the analogy case, C8 mentioned the characteristics of the idea that would be created when applied to the University of Tokyo. At this time D8 did not have a specific idea that was realistic, but C8 suggested an idea. This idea was subsequently improved by A8. In this way, the team jointly created an idea using analogical thinking, which affected later idea generation in their homework. EAP reflected this joint information processing.

**Table 6:** Key utterances in the fifth EAP section of team 8.

Index	Start time	Speaker	Utterance
t	0:55:26	D8	I wonder what it is about Pokémon Go that makes it so appealing to consumers and makes them want to play it. I feel like it is a wish to collect all the different Pokémon out there.
u	0:56:37	C8	You mean something that can be collected by getting into Tokyo University?
v	0:56:48	D8	Like turning a teacher into a Pokémon.
w	0:58:25	C8	This may sound strange, but how about partnering with several universities that also want to improve the diversity of their students, and having seminars at each university. If they attend one, they get a stamp, and if they collect all the stamps, they get something. Then we can make an opportunity to know what Tokyo University is actually doing.
x	0:59:53	B8	I wonder if this will lead to a community of students taking the entrance exam.
y	1:00:19	A8	I guess so, but if we hold an in-person event, people in rural areas won’t be able to come.
z	1:02:08	A8	How about holding a tour of the rural area like B1 Grand Prix?

The conversation was originally Japanese and translated by the first author.

The third EAP section (27 ~  40 minutes) is also related to analogical thinking. Table 7 showed the key utterances in the third section. In the analysis of the Tanita cafe case, three members C8, A8, and D8 suggested different mechanisms of attracting people (ab, ac, ad). With three members focusing on different aspects of the same case, members could find out more ways to utilize an analogy case than they could have thought of alone. This analysis indeed led to creation of new ideas (ae) that were further elaborated by multiple members (af, ag, ah). Similar to the fifth EAP section, this discussion could be understood as joint information processing for generating an idea.

**Table 7:** Key utterances in the third EAP section of team 8.

Index	Start time	Speaker	Utterance
aa	0:26:40	C8	Why was the Tanita Cafe that worked so well so attractive from the outside?
ab	0:27:50	A8	About the factors for making it attractive from the outside, I think the secret image of in-house cafeteria is one thing
ac	0:29:41	D8	And, I don’t know, the TV introduction of the company cafeteria?
ad	0:31:16	C8	The hit of a book on the menu of Tanita Cafe? I don’t know.
ae	0:31:30	C8	Then, how about publishing a book about something that only students at Tokyo University know about?
af	0:32:53	A8	If you publish a book, it won’t be like Tanita if it doesn’t become a hit, right?
ag	0:33:03	C8	Maybe you can distribute it for schools at first.
ah	0:33:30	A8	I was looking at the TripAdvisor case, and thinking of its customization for meeting customer needs. It just hit me that we can separate the information and distribute the ones that match the needs, like the book for the progressive schools, the book for the girls’ schools, and so on.

The conversation was originally Japanese and translated by the first author.

The rest of the sections showed weaker relationships with analogical thinking. The first section (0 ~  10 minutes) started with unrelated talk, and briefly talked about the possibility to utilize analogy cases for idea generation. B8 asked whether analogy cases are useful or not, and D8 and C8 answered with examples. Although these examples were not correct in terms of describing other mechanisms than attracting people, they convinced B8 to further analyze analogy cases. The team then discussed target people, but this discussion ended soon. Although the discussion about the usefulness of analogy cases contributed to subsequent analysis of analogy cases, this discussion was not the main part of this section reflecting EAP.

The second section (15 ~  19 minutes) included the discussion about two analogy cases, but many utterances were back channels or soliloquy. Although this indicated participation, it did not relate to analogical thinking.

In the fourth section (43 ~  45 minutes), the team searched for next cases for analysis together, and started to discuss the Hometown Tax Donation Program case. EAP captured the scene where everyone suggested the candidate of analogy cases to be analyzed. This discussion was also not directly related to analogical thinking.

#### 3.2.3. Team 5.

**Discussion process:** Table 8 showed the discussion topics of team 5. Team 5 spent most of the time for individual work analyzing analogy cases. They sometimes shared their analysis results to other team members, but this discussion did not last long. As a result, unlike team 4 and team 8, each member used different analogy cases for idea generation in the homework. Only one of their ideas were structurally similar to the analogy case, and two of them did not use analogy at all.

**Table 8:** Discussion topics of team 5.

Index	Start time	Topic
5.1	0:00:00	(Individual work)
5.2	0:02:04	Availability of the short report of last class
5.3	0:03:08	(Individual work)
5.4	0:20:25	How each case can be utilized
5.5	0:30:23	(Individual work)
5.6	0:36:10	Whether Mural is working
5.7	0:37:54	How each case can be utilized
5.8	0:38:49	(Individual work)
5.9	0:49:45	What to do next
5.10	0:49:55	(Individual work)

**Pattern of turn-taking:**

**Fig 9:**
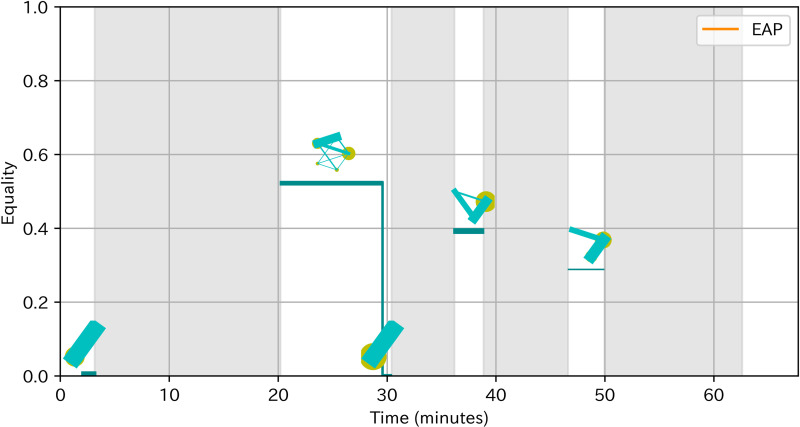
Pattern of turn-taking of team 5.

Fig 9 showed the pattern of turn-taking of team 5. Team 5 did not experience EAP sections. Even when a member shared the analysis result, other members had just appraised and did not make a constructive argument. Table 9 showed the typical discussion of the team 5. After the individual work, C5 shared his analysis result (ai), but his analysis was accepted without any elaboration from other team members (aj, ak). After a longer silence of about 10 seconds, C5 changed the topic (al). Later, D5 also shared his analysis result (am), but ended without any elaboration (an, ao).

**Table 9:** The typical discussion of team 5.

Index	Start time	Speaker	Utterance
ai	0:21:07	C5	Especially on the right side, Huis Ten Bosch, may I share? It says that in this area, there will be a full regional and local discount campaign for locals, resulting in 60% from the Kyushu area. If we reverse this structure, if we try to attract people from outside the metropolitan area, we can lower the tuition and accommodation fees from outside the metropolitan area. That’s about all I could find, to be honest.
aj	0:22:19	B5	I haven’t found anything.
ak	0:22:23	E5	Honestly, I could not understand. It was great that you came up with even one thing.
al	0:22:39	C5	I mean, it’s just too hard, you know? For strategy, I think it’s important to attract female students, rural students, and students with economic disadvantage, so I think I should find something that attracts people.
am	0:24:03	D5	Can I say one thing? I know it’s totally different from what you just said, but Hometown Tax Donation Program is working to reduce the economic disparity between regions, so I was thinking that maybe we can do something about the economic disparity between urban and rural areas by it.
an	0:24:33	C5	Which group analyzed it?
ao	0:24:35	D5	Group 4. Maybe we have to say something new, or breakthrough? I don’t remember what he said, but if we can feel the case can be used for anything, we can think about it.

The conversation was originally Japanese and translated by the first author.

#### 3.2.4. Team 7.

**Discussion process:** Because team 7’s discussion process was unorganized, we could not make a table to depict it adequately. The topics changed frequently within a short time. They generally tried to find similarities between problems of the target people and analogy cases. Their work was rather finding and checking any similarities than analyzing the cases. Their discussion was mainly about confirmation of the fact and included only a few mutual elaborations and decision making. As a result, every member used a different analogy case, and none of their ideas were structurally similar to the analogy case.

**Pattern of turn-taking:**

**Fig 10:**
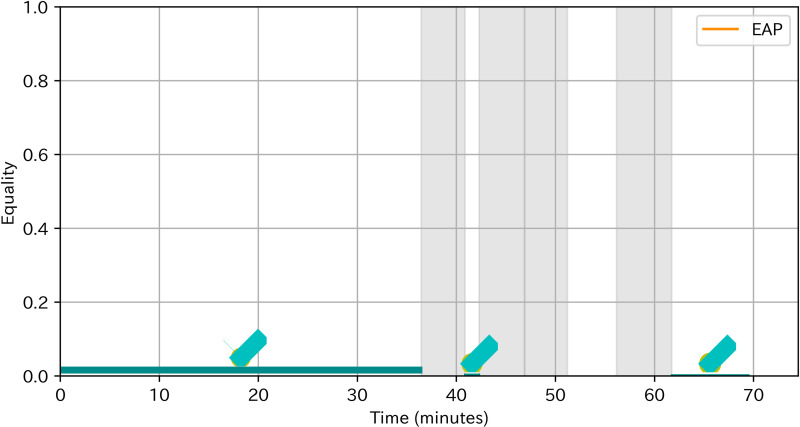
Pattern of turn-taking of team 7.

Fig 10 showed the pattern of turn-taking of team 7. Among 4 members of team 7, only two participated in the discussion. One had no utterance and the other speaker only once. Thus, equality was almost 0 throughout the discussion.

#### 3.2.5. Factors leading to equal participation: comparison between the teams.

By comparing teams with EAP sections and teams without EAP sections, and EAP sections and other sections, we found several factors leading to equal and active participation.

**Maintaining a shared understanding of what to discuss and why to discuss:** One of the characteristics of team 4 was to make consensus for each part of discussion before going to the next topic. For instance, they decided the target people (4.4) and decided the analogy case they should focus on (4.13). This consensus clarified the focus of the team, so that they could build a shared understanding of what to discuss and why to discuss in the subsequent time. This understanding could facilitate members participation by reducing cognitive load to understand others’ comments and increasing motivation to participate. Team 8 also clarified the possibility of utilizing analogy cases for idea generation in the early phase (8.2). Although the explanation was not perfectly correct, this convinced members of the importance of analyzing analogy cases. On the other hand, in team 7, one member frequently changed the topic. This made it hard for other members to keep up the topic. Also, because the topic was decided by only two members, other members might not find any significance in discussing the topic. This could be one of the reasons for no participation of two members in team 7.

**Critical comments for extending discussion:** In EAP sections, there were often objections to the current view or concerns about the idea. These critical comments made the team aware of the need for continuing discussion, which motivated members to participate. For example, in the beginning of the third EAP section of team 8, B8 pointed out a drawback of the idea of books written by students at the University of Tokyo. This comment was followed by a solution to overcome it from all other members. Similarly, in the fifth EAP section of team 4, D4 pointed out the drawbacks of the idea by comparing the created idea and the analogy case. This comment was also followed by a solution to overcome the drawback. We counted such critical comments in our data for observational analysis. For team 4, 6 out of 9 critical comments were in EAP sections, and all EAP sections included critical comments. For team 8, 5 out of 8 critical comments were in EAP sections, and three out of 5 EAP sections included critical comments. For team 5 and team 7, there were no critical comments.

**Arguing without completion:** One of the reasons for the lack of continued discussion on Team 5 could have been the norm that completed opinions should be expressed. The everyday survey revealed that several members in team 5 had tried to suggest deepening their case analysis or focusing on structural similarities, but they did not because they felt they themselves did not know how to analyze the cases. This indicated that there was a norm that they should give a complete opinion, which prevented their participation. In contrast, members in the team with EAP sections often made arguments with noticing their incompletion. For example, in team 4, C4 presented his idea with mentioning that the structural similarity was not established. Also, in Team 8, in a scene of confirming factual information about the analogy case, C8 shared his guess without knowing the truth. Although the correctness of his guess was not confirmed, this guess led further discussion and new ideas. Also, when team 8 tried to create an idea based on analogy cases, D8 did not hesitate to talk about her wild and incomplete idea. Others laughed but tried to interpret her idea. These examples suggested that making an argument, even if it was not perfect, could advance the discussion and stimulate others to improve that argument.

## 4. Discussion

Our result suggested the positive relationship between temporal equal and active participation and learning of analogical thinking in synchronous collaborative learning. Our regression analysis exhibited that the teams with more equal and active participating sections had more members who generated an idea based on structural similarity. Our observational analysis supported this finding by showcasing that joint information processing for generating ideas facilitated by equal and active participation influenced members’ individual thinking process for idea generation. Experiencing such an information processing that would have been difficult to do individually enabled the participants to acquire analogical thinking. However, our observation also demonstrated some EAP sections were indirectly or not related to learning gains. We suspected that equal and active participation contributes to learning through the mediation of joint information processing, but this relationship could also depend on other factors such as discussion topics. While measuring and utilizing equal and active participation are useful for supporting teams as a candidate for time contributing to learning gains, determining the contribution to learning gains in each case requires a further combination of other information, such as the content of the discussion.

This study has several theoretical and practical contributions. First, we empirically demonstrated the positive relationship between temporal equal and active participation and learning in synchronous collaboration. Although our result supported positive effect of equal and active participation, our result was inconsistent with existing research analyzing equal participation in all time in asynchronous collaboration [10,15,26] because our additional regression analysis revealed no correlation between learning gain and equality or activeness during the whole time. As for our case in observational study, team 5 was an example with high overall equality and less EAP sections. This team had limited participation in discussions on individual topics, but because of the rotation in participating members, the overall participation was equal. The learning gains in such a team was low in our data. Therefore, this research suggests that different phenomena may be captured depending on the measurement span of participation and future research should pay more attention to it. Additionally, measuring participation over shorter spans has revealed temporal communication dynamics such as rotating participants in discussions as team 5. Several studies have indicated that rotating participation rather demonstrates positive effects in teamwork [51,52]. These findings do not align with our observations, necessitating further investigation into the moderating factors.

In addition, our study suggested a nuanced causal relationship between equal and active participation and learning. Equal and active participation could affect learning through joint information processing, but this effect partly depended on the discussion topics.

Qualitative observations suggest that when teams engage in discussions for idea generation or analogical thinking, equal and active participation directly impacts on learning analogical reasoning. This finding is consistent with prior studies on group flow [40], where equal and active participation facilitated group flow, allowing members to share, negotiate, and elaborate on knowledge, thereby fostering knowledge creation. Conversely, equal and active participation during discussion on the discussion process or other topics did not directly produce learning gains although they could potentially indirectly influence learning gains as it can nurture shared understanding of the discussion process. Future research of equal and active participation can consider its interaction with topics for more precise understanding of its effect.

This study also listed three conditions for equal and active participation by comparing teams with EAP sections and teams without EAP sections as well as comparing EAP sections and other sections: maintaining a shared understanding of what to discuss and why to discuss, critical comments for extending discussion, and arguing without completion. Previous studies have identified a number of factors as antecedents of equal participation, such as status difference, communication comprehension, group size, and positive interdependence of task [53,54], but most of these are determined at the time of forming a team. While these are useful for grouping participants, they are not useful for increasing participation once teamwork starts. In contrast, conditions in our study are features of ongoing teamwork. Thus, they are useful for team members or educators who seek to achieve equal and active participation. Practical implications can be derived based on the findings of this study that are applicable not only to workshops for idea generation but also to a wide range of educational activities using synchronous collaborative learning. In addition to team formation based on variables related to equal participation shown in previous studies, it would be beneficial to assign a mentor to each team to observe the discussion and provide advice to encourage the three types of utterance that promote equal and active dialogue, as identified in this study. Because mentor characteristics and speech influence teamwork [55], these three types of utterance could be encouraged through prior training for team members and through instructions for teamwork. The possibility of improving learning gains through the design of teamwork, in addition to the characteristics of the learners, would be meaningful in designing inclusive educational practice.

Lastly, our study suggested the effectiveness of real-time measurement of equal and active participation. By measuring the equality and activeness of participation in real time, it is possible to estimate whether teams are experiencing effective or ineffective discussions for learning. Teams that do not experience equal and active participation can explore the causes and improve their collaboration for enhancing learning gains. Teams that experience an equal and active discussion also can improve the learning gains by reviewing the content of the discussion during equal and active participation and remembering it as a successful experience. These can be facilitated by the team on their own or by supporting outsiders such as teachers. The recent proliferation of digital tools such as Zoom and Slack has increased efforts to visualize student activities in collaborative learning. This enables researchers to analyze activities contributing to learning in detail [11,56], allows instructors to support underperforming teams in real-time, and enables students themselves to achieve more advanced collaborative learning [11,57,58]. An important issue arising in this context is the choice of indicators for visualization and various scholars particularly in the field of learning analytics have proposed unique indicators [22,59,60]. This research builds on these lines of studies by exploring the importance of temporal equal and active participation, which is a distinct process that occurs within a short time and generates new knowledge by joint information processing. Future research could more thoroughly examine the antecedents and effects of temporal equal and active discussions, as well as their relationship with learning theory in collaborative learning such as knowledge construction and knowledge creation.

While our main focus in this study was on the relationship between temporal equal and active participation and team learning gains in synchronous collaborative learning, we collected individual data on knowledge of analogical thinking, willingness to learn, interest in the workshop theme, and openness to new things through questionnaires conducted before and after the workshop, and attempted to analyze the effects of these on team learning gains in the concurrent study [43]. Although statistical analysis is still a future issue due to the limited number of participants, it was inferred that participants with higher interest in the workshop theme and openness to new things tended to achieve higher learning gains. On the other hand, it was not clear whether the willingness to learn influenced learning gains. It was clear that participants with low prior knowledge of analogical thinking had significantly lower learning gains than the other participants, but it is difficult to estimate the correlation between prior knowledge and learning outcomes at this stage.

The idea generation process in the workshop of this experiment was specifically instructed, and the subject and timing of each work during the workshop did not differ among the teams. As already mentioned, the teams were divided as evenly as possible based on the demographic data collected in advance, such as gender, high school background, and experience in creative activities. However, the characteristics of the teams would not be sufficiently equal due to the limited number of participants, and it remains to be verified with a larger sample whether these demographic data affect the correlations estimated in this study. For example, in the concurrent study [43], a descriptive analysis suggests that participants with higher scores on experience in creative activities, i.e., having participated in activities that generate new ideas such as business idea contests, have higher learning gains. In addition to equal and active participation, there are other possible factors that influence learning gains and workshop outcome in terms of utterances and actions during the workshop. Further micro-level analyses using teamwork recordings and transcripts would also be an area for future research.

Similar to most of the studies, this study had several limitations. Firstly, our result was based on data of a limited number of teams. Also, participants of our study are students in one of the top universities in Japan who generally have a high cognitive ability. Future studies can test the reliability of our findings by increasing the number of teams with more diverse participants. Another limitation is the subjectivity of our observational analysis. Though we carefully based our arguments on the fact, our point of focus and our interpretation were inevitably subjective. Thus, our results of observational analysis should be treated as hypotheses that should be tested in future studies rather than reliable findings. Lastly, there could be problems in the measurement of learning gains and equal and active participation. We measured the team learning gains by the rate of students who created ideas with structural similarity to analogy cases. However, creation of appropriate ideas does not necessarily reflect the understanding of analogical thinking. Participants could create appropriate ideas by just imitating appropriate ideas discussed in teams. Also, previous studies have measured the effects of collaborative learning on a wide range of variables, including knowledge acquisition [13], development of specific collaborative behaviors [15], and non-cognitive skills such as self-efficacy [61]. Different results may be obtained in this study if another educational objective, such as confidence in idea generation or deepness of understanding, is used in the analysis. Although we believe that our measure of team learning gains still indicates the learning effectiveness of collaborative learning, future research can develop more reliable measures. As for temporal equal and active participation, the value could change if we adopt different parameters for detecting the point of great change in communication pattern. Although this instability is inevitable, future studies can check robustness by systematically choosing multiple combinations of parameters. Also, our discussion data included back-channel and other utterances that do not advance discussion. Future studies can rule out these utterances for calculating participation equality if researchers are interested in joint information processing.

## 5. Conclusion

Collaborative learning is an essential part of 21st century learning. A growing number of formal and informal learning have incorporated collaborative learning, and the need for support learners in collaboration is continuously increasing. Simultaneously, the modern learning environment has brought big data on learner’s behavior. Information about what learners say and do in synchronous and asynchronous collaboration is becoming easier to obtain. Such data, unlike surveys, are intrusive and can be measured in real time, and thus have the potential for being used to improve the quality of collaborative learning. In supporting learners using behavioral data, the level of participation is one of the useful indicators of their current quality of collaboration. Previous studies have examined the relationship between the level of participation and learning effectiveness, as well as intervention methods to increase participation. However, the findings on temporal equal and active participation in synchronous collaboration, especially its relationship with learning gains and its antecedent, are still insufficient if one uses it to support learners.

Therefore, in this study, we qualitatively and quantitatively analyzed the relationship between temporal equal and active participation and learning gains with 10 teams that collaboratively learn analogical thinking. Only by utilizing data on micro-scale temporal communication, which has become easier to obtain due to technological advances, did we obtain evidence on the positive relationship of temporal equal and active participation in synchronous collaborative learning with learning gains. Furthermore, our observations suggested that while equal and active participation could facilitate learning through joint information processing, the effect could be direct, indirect, or absent, depending on the discussion topic and other micro-scale conditions.

The theoretical contribution of this study is that it created evidence and new empirical research questions to elaborate the theory that promoting equal and active participation enhances learning gains in light of the specific educational practice context of synchronous collaborative learning. In other words, this study opened the door to empirical research that bridges theory and practice to design effective synchronous collaborative learning with modern technologies of data collection.

Additionally, by comparing teams with more equal and active participating sections and teams without them, we proposed three antecedents of equal and active participation. Despite the limitations of this study, including insufficient sample size, subjectivity of the observational analysis, and inadequate measurement, we believe that our results provided insight about temporal equal and active participation in synchronous collaboration and about more effective ways to support collaborative learning.

## Supporting information

S1 Table Evaluation of analogy questions in pre-survey and generated ideas. (PDF)

S2 Table Data used for regression analysis. (PDF)
