# Supplementary material for: Temporal equal and active participation in synchronous collaborative learning: Antecedents and effect for learning
Source: PLoS One. 2025 Mar 24;20(3):e0318122. doi: 10.1371/journal.pone.0318122 (PMC11932484; doi:10.1371/journal.pone.0318122)
Supplement: S1 Table — (PDF) [file pone.0318122.s001.pdf]

Name	Team	Number of correct answers t	Idea evaluation of the evaluator 1	Idea evaluation of the evaluator 2	Idea evaluation of the evaluator 3
A1	1	1	structural	structural	structural
B1	1	0	superficial	superficial	superficial
C1	1	1	superficial	superficial	superficial
D1	1	2	structural	superficial	superficial
E1	1	2	structural	structural	structural
A2	2	2	superficial	structural	structural
B2	2	1	structural	superficial	superficial
C2	2	2	superficial	superficial	superficial
D2	2	2	structural	structural	structural
E2	2	3	superficial	structural	superficial
A3	3	1	superficial	superficial	superficial
B3	3	1	structural	superficial	structural
C3	3	1	superficial	superficial	superficial
D3	3	1	structural	superficial	structural
E3	3	1	structural	superficial	superficial
A4	4	1	structural	structural	structural
B4	4	1	superficial	superficial	superficial
C4	4	1	structural	structural	structural
D4	4	NA	structural	superficial	structural
A5	5	0	unrelated	unrelated	unrelated
B5	5	0	superficial	superficial	superficial
C5	5	3	superficial	structural	superficial
D5	5	0	unrelated	unrelated	unrelated
E5	5	1	structural	superficial	structural
A6	6	1	unrelated	superficial	unrelated
B6	6	0	structural	superficial	structural
C6	6	1	structural	superficial	structural
D6	6	2	structural	structural	structural
A7	7	1	unrelated	unrelated	unrelated
B7	7	0	unrelated	unrelated	unrelated
C7	7	3	unrelated	unrelated	unrelated
D7	7	3	superficial	superficial	superficial
E7	7	1	superficial	superficial	unrelated
A8	8	2	structural	structural	structural
B8	8	2	structural	structural	structural
C8	8	1	structural	structural	structural
D8	8	2	structural	structural	structural
A9	9	2	structural	structural	structural
B9	9	0	structural	superficial	superficial
C9	9	0	structural	superficial	superficial
D9	9	1	structural	superficial	structural
E9	9	1	structural	superficial	structural
A10	10	3	superficial	superficial	structural
B10	10	0	superficial	superficial	superficial
C10	10	0	unrelated	unrelated	unrelated
D10	10	0	structural	structural	structural
E10	10	2	structural	superficial	structural
