# Supplementary material for: Temporal equal and active participation in synchronous collaborative learning: Antecedents and effect for learning
Source: PLoS One. 2025 Mar 24;20(3):e0318122. doi: 10.1371/journal.pone.0318122 (PMC11932484; doi:10.1371/journal.pone.0318122)
Supplement: S2 Table — (PDF) [file pone.0318122.s002.pdf]

Team	Rate of EAP section	Team learning gain	Average number of correct answers to analogy questions	Rate of EAP section with stricter threshold	Rate of EAP section with looser threshold	Rate of only equal participating section	Rate of only active participating sections	Equality in all time	Activeness in all time
1	0.24	0.40	1.20	0.16	0.26	0.32	0.40	0.60	3.31
2	0.06	0.40	2.00	0.04	0.06	0.22	0.07	0.63	1.23
3	0.20	0.40	1.00	0.03	0.30	0.60	0.21	0.62	2.98
4	0.40	0.75	1.00	0.26	0.69	0.53	0.57	0.66	4.22
5	0.22	0.20	0.80	0.14	0.35	0.44	0.27	0.75	2.61
6	0.26	0.75	1.00	0.16	0.29	0.45	0.32	0.67	2.73
7	0.02	0.00	1.60	0.01	0.06	0.09	0.61	0.36	3.68
8	0.68	1.00	1.75	0.58	0.75	0.91	0.79	0.72	5.04
9	0.13	0.60	0.80	0.13	0.13	0.14	0.34	0.47	2.64
10	0.01	0.40	1.00	0.01	0.01	0.30	0.47	0.47	2.84
